# Role of Radiotherapy in the Era of Novel Immune‐Based Treatment Strategies for Multiple Myeloma

**DOI:** 10.1002/hon.70186

**Published:** 2026-03-15

**Authors:** Tom Schlusemann, Evgenii Shumilov, Gabriele Reinartz, David Rene Steike, Sebastian Lohmann, Stefan Gravemeyer, Matthias Stelljes, Georg Lenz, Hans Theodor Eich, Michael Oertel

**Affiliations:** ^1^ Department of Radiation Oncology University Hospital Muenster Muenster Germany; ^2^ Department of Medicine A, Hematology, Oncology and Pneumology University Hospital Muenster Muenster Germany

**Keywords:** bispecific antibodies, chimeric antigen receptor, immunotherapy, multiple myeloma, radiotherapy

## Abstract

Radiotherapy (RT) constitutes an important treatment modality in multiple myeloma (MM), traditionally used for the management of symptomatic osteolytic and extramedullary lesions. Objectives of RT are symptom alleviation and prevention of complications such as neurological deficits and pathological fractures. Over the past decade, systemic therapy (ST) for MM has undergone substantial advancements, notably with the incorporation of monoclonal antibodies in frontline regimens, as well as the emergence of bispecific antibodies and chimeric antigen receptor T‐cells (CAR‐T) for relapsed and refractory disease. These therapeutic innovations have introduced new clinical challenges and expanded the indications for RT, including its role as bridging therapy prior to CAR‐T therapy, salvage therapy following CAR‐T failure, and in the management of oligoprogressive and/or extramedullary disease. The integration of novel systemic agents prompts critical questions regarding synergistic interactions, feasibility, efficacy, and tolerability of combined RT and ST approaches. Herein, we present a literature review summarizing current evidence stratified by systemic treatment modalities, and discuss the clinical implications within the contemporary therapeutic landscape of MM.

## 1 Introduction

Multiple Myeloma (MM) is a subtype of plasma cell neoplasms, characterized by the monoclonal proliferation of plasma cells in the bone marrow and the secretion of monoclonal immunoglobulins [1]. Systemic therapy (ST) options for MM have rapidly evolved over the past few years, with anti‐CD38 directed monoclonal antibodies (MAbs) such as daratumumab and isatuximab forming the backbone of first‐line therapy [2, 3, 4, 5, 6, 7, 8] alongside the introduction of bispecific antibodies (BsAbs) [9, 10, 11] and chimeric antigen receptor T‐cells (CAR‐T) [12, 13] for relapsed and refractory multiple myeloma (RRMM). Consequently, patients may be offered an extended number of therapeutic lines compared to recent years resulting in prolonged overall survival (OS) [14, 15]. Traditionally, radiotherapy (RT) has been used to target osteolytic and extramedullary lesions in MM, which occur in approximately 80% of patients at initial diagnosis and are often associated with pain, neurological deficits, compression or pathological fractures [16]. While solitary osseous or extramedullary plasmacytomas may be treated with curative intent using RT alone [17, 18], the systemic nature of MM necessitates on a combination of RT and ST to achieve effective disease and symptom control. With the rapid advancement of ST, new indications for RT have emerged, including bridging to CAR‐T therapy, salvage RT after CAR‐T failure, application of RT in combination with BsAbs for induction of response and the control of subsequent oligoprogressive disease.

## 2 Evolution of Therapy in Multiple Myeloma

Over the last few decades, treatment options for MM have significantly advanced, leading to improved progression‐free survival (PFS) and OS rates [14, 15]. From conventional cortisone‐containing chemotherapy approaches including melphalan and/or doxorubicin, bendamustine and vincristine [19, 20], treatment strategies have progressed with the implementation of immunomodulatory drugs such as thalidomide and lenalidomide, as well as proteasome inhibitors like bortezomib, ixazomib or carfilzomib [21, 22, 23, 24].

The traditional role of RT in the management of MM has been the treatment of symptomatic osteolytic and extramedullary lesions, as well as the management of impending fractures. Patients often present with rapidly progressive disease requiring urgent initiation of ST and concurrent RT due to symptomatic lesions and the risk of fractures. The combination of RT and ST in MM may offer a therapeutic synergism and raises important questions regarding safety and feasibility, particularly concerning hematologic toxicities and bone marrow function during and after combined therapy. Several studies have evaluated the safety and feasibility of RT administered concurrently with ST in the pre‐CAR‐T and BsAb era, mainly focusing on immunomodulatory drugs and proteasome inhibitors. Shin and colleagues demonstrated no increased risk of non‐hematologic toxicities in a small cohort of 39 patients receiving concurrent ST during RT, 17 of whom had received at least one biological agent [25]. Resende Salgado and colleagues reported similar findings in a cohort of 130 patients, including 91 who received concurrent RT and ST, with no significant difference in acute (within 4 weeks after RT completion) or subacute (within 6 months after RT completion) non‐hematologic toxicities. Additionally, no significant differences were observed in white blood cell count, platelet levels, or hematocrit before and after RT [26]. Guerrini and colleagues observed a slight increase in non‐hematologic toxicities during RT when combined with ST, primarily grade 1 and 2 events according to Common Terminology Criteria for Adverse Events (CTCAE) [27]. However, this trend was no longer evident at one and 3 months following RT completion [28]. Our recent study analyzed hematological and non‐hematological toxicities in a cohort of 82 patients, of whom 53 received concurrent RT and ST. While no significant differences in hematologic toxicities were found during RT, a significant increase in high‐grade (grade 3 or 4) hematologic toxicities occurred in the multimodality treatment group after completion of RT (within 3 months). A significant increase in thrombocytopenia and leukocytopenia was shown for larger treatment volumes including at least five osseous sites. Regarding non‐hematologic toxicities, no significant differences occurred comparing RT alone and RT in combination with ST [29].

The implementation of modern therapeutic approaches has led to new indications for RT:

– bridging RT before CAR‐T therapy to reduce disease burden, particularly extramedullary disease (EMD), and potentially improve prognosis
– salvage RT after CAR‐T failure with possible immunomodulatory effects and T‐cell activation to trigger a systemic disease response
– RT for the treatment of EMD and/or oligoprogressive disease under ongoing treatment with BsAbs

Consequently, there is a paucity of evidence concerning the efficacy, potential synergism, and safety of combining modern ST and RT.

This prompted our group to perform a selective literature review using PubMed with the search string 'multiple myeloma AND radiotherapy AND (CAR‐T OR bispecific antibodies OR CD38‐antibodies)' covering the period from January 1, 2010, to November 1, 2025, to review the current evidence. Only English‐language studies were included. Additional relevant articles were identified through a manual review of references cited in the selected literature.

The gained insights are grouped according to systemic medication and discussed in light of the clinical context whenever possible.

### 2.1 Radiotherapy and Monoclonal Antibodies

MAbs are a backbone of treatment in MM. Daratumumab and isatuximab target CD38 [2, 6], while elotuzumab targets the antisignaling lymphocytic activation molecule F7 (SLAM F7) [30]. Daratumumab was first approved by the FDA for the treatment of RRMM in November 2015, initially as monotherapy and later, in November 2016, as part of triple therapy in combination with lenalidomide/dexamethasone or bortezomib/dexamethasone. Today, CD38‐directed antibodies such as daratumumab and isatuximab are considered essential components of first‐line MM treatment.

In the United States, approval for first‐line use of daratumumab in transplant‐ineligible patients was granted in June 2019, based on the compelling results of the MAIA trial [6]. Approval for transplant‐eligible patients followed in September 2019, supported by results from the CASSIOPEIA trial [8]. Several recent studies have established quadruplet regimens as the new standard of care, incorporating anti‐CD38–targeted MAbs (daratumumab in PERSEUS and CEPHEUS; isatuximab in GMMG‐HD7 and IMROZ) in combination with bortezomib, lenalidomide and dexamethasone, followed by MAb plus lenalidomide and dexamethasone–based maintenance. This treatment paradigm applies to both transplant‐eligible patients (PERSEUS and GMMG‐HD7) and transplant‐ineligible patients (CEPHEUS and IMROZ) [2, 3, 4, 5].

The aforementioned analysis by Resende Salgado and colleagues evaluating the safety of concurrent RT and biological agents, included a small subcohort of 25 patients who received daratumumab within a timeframe spanning from one month before to one month after RT. In this subgroup acute grade 1 or 2 side effects (occurring within 4 weeks after completion of RT) were observed in 50% of patients, while 22% experienced subacute side effects (occurring between 4 weeks and 6 months post‐RT). No high‐grade toxicities were observed during treatment [26].

To the best of our knowledge, no additional studies have systematically investigated the concomitant use of MAbs and RT in the treatment of MM.

### 2.2 Radiotherapy and CAR‐T

CAR‐T cells are genetically modified T‐cells designed to target specific antigens, in case of MM directed against the B‐cell maturation antigen (BCMA). Implementation of CAR‐T cells in the treatment of MM represents an important option for patients with RRMM. In MM, two CAR‐T products—idecabtagene vicleucel (ide‐cel) and ciltacabtagene autoleucel (cilta‐cel)—are widely used and have been evaluated in major Phase 3 trials. Ide‐cel was assessed in the KarMMa‐3 trial including 386 patients with RRMM after receiving two to four prior therapy lines. In a 2:1 randomization ide‐cel showed a significantly prolonged PFS compared to standard‐treatment‐regimens (median 13.3 vs. 4.4 months; hazard ratio 0.49, *p* < 0.001) and higher overall response rates (ORR) compared to standard treatment regimens (71% vs. 42%; *p* < 0.001) [13]. Similar results were reported for cilta‐cel in the CARTITUDE‐4 trial investigating the effect of cilta‐cel versus standard regimens in lenalidomid‐refractory MM. With a median follow‐up (FU) of 15.9 months PFS was significantly higher in the cilta‐cel versus standard regimens group (median not reached vs. 11.8 months; hazard ratio, 0.26; *p* < 0.001). Again, ORR was higher with CAR‐T therapy (85% vs. 67%; *p* < 0.001) [12]. Updated, but not yet fully published, data (median FU 33.6 months) presented at the International Myeloma Society Annual Meeting 2024 demonstrated a consistent and significant PFS benefit (median PFS not reached vs. 11.8 months; 30‐month PFS 59% vs. 26%).

#### 2.2.1 Bridging RT Before CAR‐T

The manufacturing process of CAR‐T cells following leukapheresis requires several weeks. The KarMMa‐3 trial reports a median time from leukapheresis to reinfusion of 49 days [13], CARTITUDE‐4 indicates a median time of 79 days [12]. While these data could be prolonged due to the COVID‐19 pandemic, real world data suggest an interval of at least 4 weeks, differing between CAR‐T products [31, 32]. Patients awaiting CAR‐T therapy represent a particularly vulnerable group with a high risk of disease progression and poor outcomes. Furthermore, higher toxicity rates of CAR‐T therapy—particularly the risk of cytokine release syndrome (CRS) and immune effector cell‐associated neurotoxicity syndrome (ICANS)—are associated with a higher disease burden at the time of CAR‐T administration [33, 34, 35]. Effective bridging strategies are crucial to prevent uncontrolled disease progression and complications that might ultimately render CAR‐T therapy unfeasible [36]. These strategies may include ST, RT, or a combination of both. In the CARTITUDE‐4 trial, bridging ST was mandated as a key component of the protocol for patients in the cilta‐cel arm. In contrast, the KarMMa‐3 trial allowed bridging ST at investigator's discretion, with 86% of patients ultimately receiving it. Although palliative RT was not explicitly prohibited during study treatment in either trial, neither KarMMa‐3 nor CARTITUDE‐4 provides data on its actual use, particularly as part of bridging therapy.

Patients with EMD represent a highly vulnerable subgroup in RRMM, with increasing evidence indicating that EMD constitutes a negative prognostic factor in the context of CAR‐T therapy. A real‐world analysis by Hansen and colleagues evaluated 159 patients, of whom 76 (48%) had EMD. In this subgroup ORR was significantly lower compared with patients without EMD (78% vs. 90%; *p* = 0.03) [37]. Similar findings were reported by Dima and colleagues in a cohort of 152 patients and by Zanwar and colleagues in 351 patients, with EMD consistently associated with inferior ORR, PFS, and OS [38, 39]. Dima and colleagues further provided detailed data on relapse patterns: 32 of 47 patients in the EMD group experienced disease progression, of whom 24 had EMD‐associated relapse (12 with EMD only, 12 with combined biochemical relapse). In 15 patients, EMD relapse occurred at the same site as prior to CAR‐T therapy, with seven patients relapsing exclusively at this location. Zanwar and colleagues further analyzed the impact of RT to all sites of EMD prior to CAR therapy, observing a trend toward improved PFS (median 6.9 vs. 4.3 months; *p* = 0.77) and OS (hazard ratio 0.44; *p* = 0.056; median OS not reported). In case of EMD bridging RT may present a promising approach to improve outcomes and control rates.

The first report on bridging RT in MM was presented by Manjunath and colleagues, describing four patients who received RT as a bridging therapy—two due to bone pain and two due to neurological deficits associated with plasmacytomas. The median dose was 22 Gy (range 8–30 Gy), delivered in three to eight fractions, with a median interval of 25 days from RT to CAR‐T therapy (range 18–35 days). No RT‐related high‐grade toxicities were observed; only one patient experienced low‐grade toxicities (grade 1 fatigue and alopecia) related to RT. A cohort of patients who had not received bridging RT or any RT within 1 year before apheresis and a cohort who had received RT within 1 year before apheresis were used as control groups. There was no significant increase in the incidence of CRS or neurotoxicity for these patients compared to those who did not receive bridging RT. An overview of the study is provided in Table 1. However, no data on local control following RT were documented for this small cohort [40].

**TABLE 1:** Overview of the data presented by Manjunath and colleagues on bridging radiotherapy (RT) [40]: Group A—no RT within 1 year before apheresis; Group B—RT within 1 year; Group C—bridging RT.

	Group A (*n* = 13)	Group B (*n* = 8)	Group C (*n* = 4)
Bridging ST	11 (85%)	6 (75%)	4 (100%)
PR or better^a^	7 (54%)	3 (38%)	2 (50%)
Grade 3 hematologic toxicities^b^	10 (77%)	2 (25%)	2 (50%)
Grade 4 hematologic toxicities	8 (62%)	5 (63%)	1 (25%)
Grade 2–4 neurotoxicity	3 (23%)	4 (50%)	1 (25%)
Grade 2–4 CRS	11 (85%)	8 (100%)	3 (75%)

Abbreviations: CRS, cytokine release syndrome; *n*, number of patients; PR, partial response; ST, systemic therapy.

^a^ based on the International Myeloma Working Group criteria.

^b^ Toxicities were graded per CTCAE v4.0 except for CRS (graded per University of Pennsylvania CRS Grading System).

A retrospective analysis conducted by Ababneh and colleagues included data from 13 patients who received RT as either bridging or post CAR‐T salvage therapy. Nine patients underwent bridging RT to 17 sites, all of which were treated with palliative intent. The median RT dose was 20 Gy (range 4–24 Gy), administered in a median of five fractions (range 1–12 fractions) to 14 osseous sites and three extramedullary manifestations. RT demonstrated excellent local control rates, with 82% of sites showing a complete response (CR) and 18% a partial response (PR). No RT‐related high‐grade toxicities were reported in the study with low‐grade toxicities primarily including fatigue, headache, pain aggravation, dermatitis, and dysphagia. No data on long‐term outcomes of irradiated lesions were provided in this analysis [41].

Two additional abstracts on bridging RT were presented at the 2025 Annual Meeting of the American Society of Radiation Oncology (ASTRO). In a cohort of 51 patients, seven of whom received bridging RT with 20 Gy in 2–4 Gy fractions, Daniels and colleagues demonstrated high local control rates and no evidence of aggravated toxicity with respect to CRS or ICANS [42]. Perez and colleagues reported on 15 patients receiving bridging RT (defined as RT administered within 90 days prior to CAR‐T therapy) and analyzed outcomes and toxicity parameters with a particular focus on patients with EMD. Patients with EMD showed a trend toward inferior PFS (median 6.9 vs. 10.1 months; *p* = 0.06). Interestingly, within the subgroup of patients with EMD, those who received comprehensive bridging RT to all EMD sites demonstrated superior PFS (6.8 vs. 4.7 months; *p* = 0.05). No local failures in previously irradiated lesions were observed [43]. Despite these encouraging findings, limitations arise from the not yet fully published nature of both analyses and the lack of detailed information regarding patient and lesion characteristics.

**FIGURE 1:**
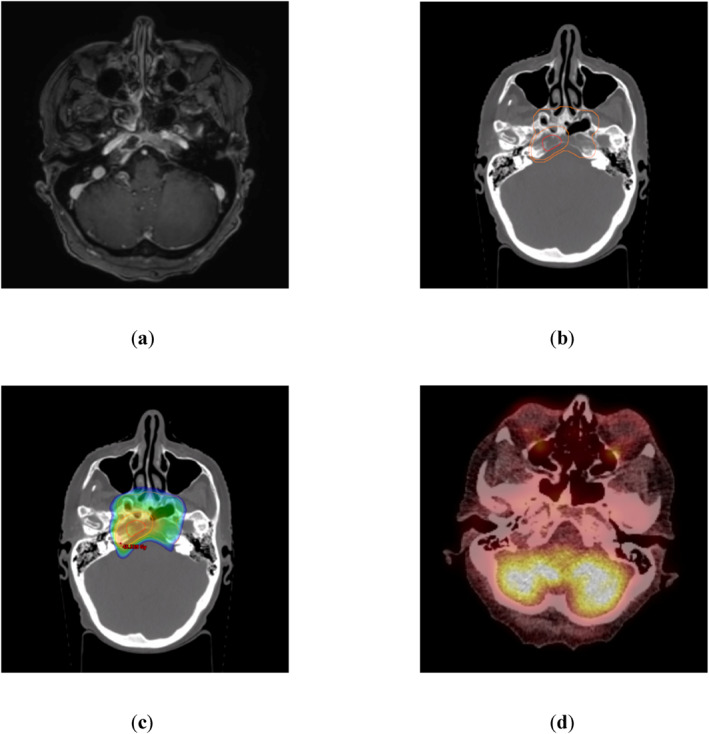
Bridging radiotherapy (RT) for a 71‐year‐old woman presenting with serological progression and abducens paresis due to a lesion in the clivus under bridging therapy with isatuximab, pomalidomide and dexamethasone prior to chimeric antigen receptor T‐cell (CAR‐T) therapy. Interdisciplinary tumor board voted for modified bridging strategy with talquetamab and concomitant local RT of clival lesion. No more lesions were detected in low‐dose computer tomography (CT) (a) Magnetic resonance imaging (MRI) showing a T1‐contrast‐enhancing lesion in the right clivus (b) Planning‐CT indicating the MRI‐based Gross Tumor Volume (red) and Planning Target Volume (orange). Cone‐down‐technique was used with a sequential boost. (c) 95%‐isodose as color‐wash delineation based on a 6 MV intensity‐modulated‐radiotherapy‐plan using volumetric‐arc‐technique [prescribed dose 36 Gy in fractions of 2 Gy with sequential boost to 44 Gy (orange contour)] (d) Positron emission tomography‐CT scan 30 days after completion of RT and before planned CAR‐T therapy revealing no metabolic activity. At the most recent follow‐up, 12 months after CAR‐T therapy, a sustained complete remission was documented.

To conclude, there is a lack of prospective trials investigating the optimal bridging regimen due to the high complexity of patients with RRMM. An example for bridging RT from our institution is presented in Figure 1.

#### 2.2.2 Salvage RT After CAR T

Despite the impressive PFS data reported by KarMMa‐3 and CARTITUDE‐4, a significant proportion of patient's experience relapse and disease progression following CAR‐T therapy. Relapse patterns can be classified into systemic disease progression and oligoprogressive disease, characterized by recurrent or newly emerging plasmacytomas.

In cases of systemic disease progression, RT serves as part of a multimodal treatment approach for symptomatic plasmacytomas. However, in oligoprogressive disease without systemic progression, salvage RT alone may be an effective strategy to delay the need for a change in ST and even enhance systemic antitumor activity.

A case report by Smith and colleagues shows evidence of a RT‐triggered abscopal‐like response after CAR‐T failure. Only a few days after receiving CAR‐T therapy, the patient developed malignant spinal cord compression and increased M‐protein levels. Dexamethasone was administered and magnetic resonance imaging (MRI) revealed spinal cord compression in the thoracic spine and multiple cerebral epidural masses with a midline shift. The patient received RT to the upper thoracic spine (20 Gy in 5 fractions), followed sequentially by whole‐brain RT (same dosage). MRI of the irradiated sites, performed 4 weeks after RT, revealed near‐complete resolution of disease. Interestingly, M‐protein levels had declined to undetectable levels by 7 weeks following CAR‐T transfusion. Moreover, whole‐body positron emission tomography—computer tomography (PET‐CT) imaging at 8 weeks post‐infusion demonstrated a CR, including the resolution of numerous disease sites located outside the RT‐field. Serological monitoring after CAR‐T administration and RT revealed a delayed CRS‐like reaction and expansion of a new T‐cell receptor repertoire after completion of RT, suggesting synergy between RT and CAR‐T therapy [44]. Ababneh and colleagues reported on eight patients who received salvage RT at 12 sites, with a median time to first RT administration of 4.2 months after CAR‐T therapy. All 12 sites were treated with palliative intent, with 50% presenting as osseous lesions and 50% as extramedullary plasmacytomas. Local in‐field control rates were excellent, with CR observed in three irradiated lesions (25%) and PR in nine irradiated lesions (75%). Notably, no high‐grade RT‐related toxicities were reported [41].

**FIGURE 2:**
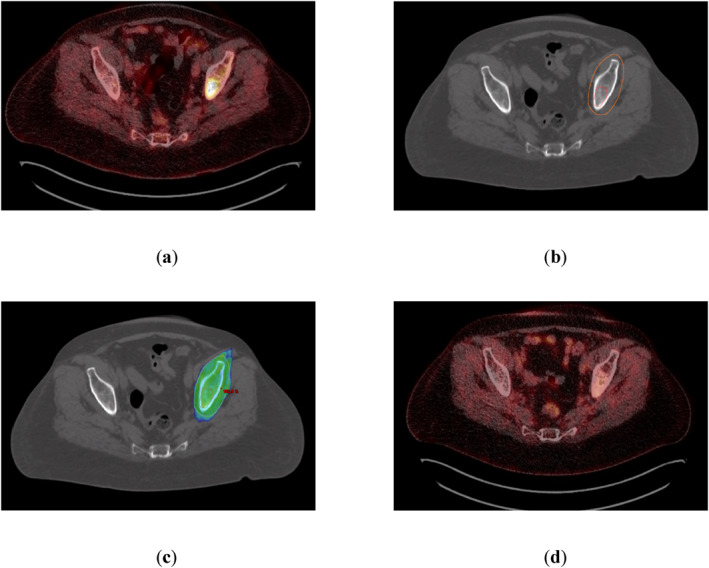
Salvage radiotherapy (RT) for a 61‐year‐old woman presenting with oligoprogressive disease in the left iliac bone on a Positron emission tomography‐computer tomography (PET‐CT) scan 30 days after chimeric antigen receptor T‐cell (CAR‐T) therapy. No more lesions were detected. Serological the patient showed a very good partial response. Interdisciplinary tumor board voted for local RT. (a) PET‐CT scan performed 30 days after CAR‐T therapy reveals a hypermetabolic lesion in the left iliac bone (standard uptake value 15; Deauville‐Score 5). (b) Planning‐CT indicating the PET‐CT‐based Gross Tumor Volume (red) and Planning Target Volume (orange). (c) 95%‐isodose as color‐wash dose distribution based on a 15 MV intensity‐modulated‐radiotherapy‐plan using 5 fields (prescribed dose 35 Gy in fractions of 2.5 Gy) (d) PET‐CT scan performed 30 days after completion of RT showing a complete metabolic remission.

However, in case of MM evidence on salvage RT is sparse and further investigations are needed to define optimal indication and timing for Salvage RT after CAR T therapy. A case of salvage RT after CAR‐T therapy due to oligoprogressive disease is presented in Figure 2.

### 2.3 Radiotherapy and Bispecific Antibodies

BsAbs represent a pivotal novel treatment option for patients with heavily pretreated MM. These antibodies feature two binding domains—one targeting a tumor cell antigen and the other binding to an immune cell antigen. By bridging tumor and immune cells, BsAbs facilitate immune cell recruitment, activation, and subsequent cytotoxicity [45].

Currently, three BsAbs have been approved for the treatment of heavily pretreated MM. Teclistamab and elranatamab target BCMA and bind to CD3 on T cells, while talquetamab is directed against GPRC5D (G protein‐coupled receptor, class C, Group 5, member D) and CD3. The approval of BsAbs in MM is based on single‐arm Phase 1/2 trials, which have demonstrated an ORR ranging from 61% to 74% [9, 10, 11].

The administration of teclistamab was investigated in the Phase 1/2 MajesTEC‐1 trial. Among 165 patients treated with teclistamab, the ORR was 63%, with a CR or better achieved in 39% of patients and median PFS of 11.3 months [10].

Elranatamab was evaluated in the Phase 2 MagnetisMM‐3 trial, in which 123 patients received the drug. The ORR was 61%, with a CR or better in 35% of patients. Median PFS had not been reached at the time of analysis, with a median FU of 14.7 months and a 15‐month PFS of 50.9% [11].

Talquetamab was assessed in the Phase 1/2 MonumenTAL‐1 trial and administered to 537 patients either intravenously or subcutaneously at various dose levels. ORR ranged from 67% to 74%, with CR or better observed in 33%–41% of patients [9].

Data on the combination of GPRC5D‐ and BCMA‐directed BsAbs were reported from the phase 1b/2 RedirecTT‐1 trial, in which 94 patients received both talquetamab and teclistamab. The ORR was 78% across all dose levels and 80% with the recommended Phase 2 regimen, with CR or better in 48% and 52% of patients, respectively. However, the combination was associated with high toxicity rates, with grade 3 or 4 toxicities occurring in 96% of patients [46].

As already observed in the context of CAR‐T therapy, EMD plays a critical prognostic role in RRMM, and the activity of BsAbs in this subgroup is essential for assessing therapeutic effectiveness. Available data consistently indicate inferior outcomes and reduced efficacy of BsAbs in patients with EMD.

For BCMA‐directed BsAbs, the MajesTEC‐1 trial reported a markedly lower ORR in patients with EMD (36% vs. 69%; no *p*‐value provided) [10]. Comparable findings were observed with elranatamab in the MagnetisMM‐3 trial, where ORR was significantly reduced in patients with EMD [11].

Similar results have been described for the GPRC5D‐directed talquetamab. In both dosing cohorts, ORR was substantially lower in patients with EMD (weekly: 82% vs. 48%; every 2 weeks: 80% vs. 41%) [9].

For the combination of GPRC5D‐ and BCMA‐directed BsAbs, the RedirecTT‐1 trial reported an ORR of 88% in patients without EMD compared with 59% in those with EMD [46].

**FIGURE 3:**
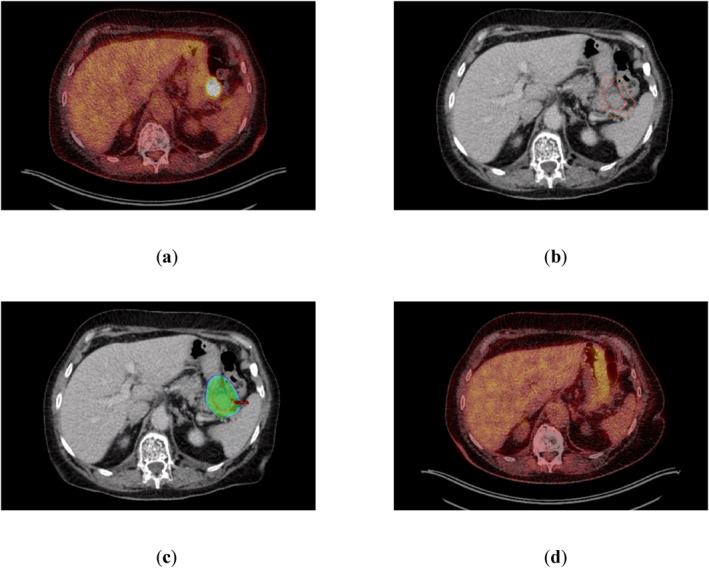
Salvage radiotherapy (RT) for a 63‐year‐old and heavily pretreated woman under ST with teclistamab presenting a paragastric lesion on a Positron emission tomography‐computer tomography (PET‐CT) scan. No additional lesions were detected. The interdisciplinary tumor board recommended local RT with continuation of teclistamab. (a) PET‐CT scan demonstrating a hypermetabolic paragastric lesion (standard uptake value 9.7; Deauville‐Score 5). (b) Planning‐CT indicating the PET‐CT‐based Gross Tumor Volume (red) and Planning Target Volume (orange). (c) 95%‐isodose as color‐wash dose distribution based on a 6 MV intensity‐modulated‐radiotherapy‐plan using 5 fields (prescribed dose 30.6 Gy in fractions of 1.8 Gy) (d) PET‐CT scan 90 days after completion of RT demonstrating a complete metabolic remission. Sustained serological complete remission was documented 6 months after completion of RT.

In this context, RT may represent a valuable therapeutic strategy for targeting EMD or managing oligoprogressive EMD during ongoing BsAb therapy, particularly in heavily pretreated patients with limited remaining treatment options. An illustrative example of oligoprogressive EMD treated with RT during ongoing teclistamab therapy is shown in Figure 3.

Limited data are available on the safety and efficacy of concurrent RT and BsAbs. Not yet fully published evidence was presented by Ababneh and colleagues at the 2025 Annual Meeting of the ASTRO. 26 patients (seven with large‐B‐cell lymphoma and 19 with MM) received RT to 44 lesions within a time window from 3 months before initiation up to 3 months after completion of BsAbs. In 19 lesions, RT was administered concurrently with BsAb treatment. With a median FU of 6 months, local control rates were excellent (CR 26%; PR 74%), and no high‐grade RT‐related toxicities or high‐grade CRS/ICANS were observed [47].

### 2.4 Radiotherapy and PD‐1/PD‐L1‐Antibodies

Despite their widespread use in solid tumors, the role of checkpoint inhibitors in hematologic malignancies remains a subject of ongoing debate. While certain subtypes—such as Hodgkin lymphoma and primary mediastinal large B‐cell lymphoma—have shown clear therapeutic benefit from immune checkpoint inhibition [48, 49], their role in the treatment of MM patients remains uncertain. Several studies investigating the combination of traditional MM‐directed treatment regimens with pembrolizumab were placed on hold due to safety concerns, including increased toxicity and unclear benefit in RRMM (KEYNOTE‐183 and KEYNOTE‐023) [50, 51] as well as in first‐line treatment (KEYNOTE‐185) [52]. Similar results were observed with nivolumab, evaluated in the CheckMate 602 trial, in which patients with RRMM were randomized to receive pomalidomide/dexamethasone (Pd), nivolumab plus Pd, or nivolumab plus elotuzumab plus Pd. The addition of nivolumab did not translate into a clinical benefit, while being associated with an increased toxicity profile and higher rates of treatment discontinuation due to adverse events [53]. Nevertheless, checkpoint inhibitors and especially combination with concurrent RT are investigated in clinical trials based on experimental results postulating an immunomodulatory effect for RT [54, 55, 56]. Deng and colleagues demonstrated an upregulation of programmed death‐ligand 1 (PD‐L1) following high‐dose single‐fraction RT (12 Gy in one fraction) in mice models, with therapeutic synergism of combinational therapy. This enhanced effect was shown to be mediated via a cytotoxic T‐cell–dependent mechanism and associated with a reduction in local levels of immunosuppressive, tumor‐infiltrating myeloid‐derived suppressor cells. Notably, the combination of high‐dose single‐fraction RT and anti–PD‐L1 treatment also elicited responses in distant, non‐irradiated tumors—a phenomenon not observed in treatment arms receiving either RT or PD‐L1 inhibition alone [54]. A Phase 2 trial published by Khan and colleagues evaluated the safety and efficacy of pembrolizumab in combination with single‐fraction RT (8 Gy) in RRMM. RT was administered to symptomatic and/or progressive osseous and extraosseous lesions. No acute high‐grade toxicities attributable to RT were observed in this study. Only one patient experienced a grade 3 toxicity within 3 months after RT, presenting with a pembrolizumab‐associated autoimmune fever. No grade 4 toxicities or treatment‐related deaths were observed. An overall response was seen in seven out of 25 patients (three with PR, two with very good partial response, and two with CR). An abscopal response (AR) in non‐irradiated lesions was observed in five patients (20%). Interestingly, three of seven patients (43%) who had previously received CAR‐T therapy exhibited an AR, compared to only two of 18 patients (11%) without prior CAR‐T administration [57]. These findings markedly surpass the outcomes of nivolumab monotherapy in RRMM reported by Lesokhin and colleagues in 2016, which demonstrated an ORR of only 4%, with stable disease observed in 63% of patients. Notably, one patient with SD after nivolumab initiation developed a delayed response after RT to a rib lesion. Following the initiation of nivolumab, the patient achieved stable disease for nearly 2 years before developing serological progression and a symptomatic rib lesion. Nivolumab was temporarily withheld for approximately 1 month during the administration of RT to the symptomatic site. Upon completion of RT, nivolumab was reinitiated and continued for an additional 2 months, during which the patient achieved a CR. Due to protocol restrictions nivolumab was discontinued 2 months after reinitiation, marking the end of the predefined 2‐year treatment course. Fourteen months after cessation of therapy, the patient remained in remission [58].

### 2.5 Dose Finding and Fractionation in the Modern Treatment Era

The optimal dosage and fractionation schemes for MM remain a widely discussed topic and are currently under ongoing investigation. Various RT regimens have been reported in the literature, ranging from single‐fraction doses of 4–8 Gy to normofractionated schedules delivering up to 50 Gy. Guidelines published by the International Lymphoma Radiation Oncology Group (ILROG) recommend the use of hypofractionated regimens with total doses ranging from 8 to 30 Gy [59]. The COVID‐19 pandemic, with its associated strain on healthcare resources, further accelerated the shift toward shortened, low‐dose RT regimens [60, 61]. For uncomplicated lesions—defined by the absence of fracture risk, prior orthopedic fixation, reirradiation and epidural involvement—Price and colleagues found no significant differences in analgesic response between low‐dose RT (with an equivalent dose in 2 Gy fractions < 12 Gy) and higher‐dose treatments [62]. Currently, a multi‐institutional study led by ILROG is investigating the efficacy of ultra‐low‐dose RT (2 × 2 Gy or 1 × 4 Gy) for the palliation of pain in patients with uncomplicated osseous lesions. Interim results from the first 40 patients demonstrate high pain response rates (48% CR, 38% PR) and low reirradiation rates of 18% [63]. In cases of complicated lesions, the need for longer‐course and moderately hypofractionated RT regimens remain, being associated with higher rates of motor function recovery in patients with myeloma‐associated spinal cord compression [64], greater degrees of recalcification [65, 66], and more pronounced improvements in quality of life [67]. This discussion becomes even more complex in the era of modern immunotherapeutic agents, due to the immunomodulatory effects of RT, which can be either immunoenhancing or immunosuppressive, potentially reducing the efficacy of immunotherapy. While both preclinical and clinical data increasingly support the use of hypofractionated radiation regimens [54, 55, 57], more evidence is needed to determine the optimal dosage, fractionation schedules, and timing of RT—whether administered concurrently or sequentially—and these aspects continue to be the focus of ongoing investigations.

## 3 Conclusions

Systemic treatment modalities have evolved rapidly in recent years, with the introduction of MAbs in first‐line treatment and the successful use of CAR‐T therapy and BsAbs in RRMM. Nevertheless, RT remains a pivotal treatment modality in the management of MM. Following the advent of modern immunotherapies, the indications for RT have expanded from palliative purposes in painful or complicated lesions to bridging approaches prior to CAR‐T therapy, salvage RT after CAR‐T failure and managing extramedullary and/or oligoprogressive disease under ongoing BsAb treatment. Despite the rapid advancements in ST, little is known about the safety and feasibility of combined treatment approaches. Further trials are required to enhance the current evidence base regarding the integration of modern and personalized ST with RT, as well as to evaluate the efficacy of multimodality treatment strategies.

## Author Contributions

T.S. and M.O. conceptualized the review, finalized the manuscript, and provided overall supervision of the panel's activities and contributions. E.S., G.R., D.R.S., S.L., S.G., M.S., G.L. and H.T.E. contributed to the preparation of the manuscript through active discussion, participated in revising the draft, and approved the final version.

## Conflicts of Interest

G.L. received research grants not related to this manuscript from AGIOS, AQUINOX, AstraZeneca, Bayer, Celgene, Gilead, Janssen, MorphoSys, Novartis, F. Hoffmann‐La Roche Ltd, and Verastem. G.L. received honoraria not related to this manuscript from ADC Therapeutics, AbbVie, Amgen, AstraZeneca, Bayer, BeiGene, BMS, Celgene, Constellation, Genase, Genmab, Gilead, Hexal/Sandoz, Immagene, Incyte, Janssen, Karyopharm, Lilly, Miltenyi, MorphoSys, MSD, NanoString, Novartis, PentixaPharm, Pierre Fabre, F. Hoffmann‐La Roche Ltd, and Sobi.

## Data Availability

The authors have nothing to report.
